# Immune checkpoint inhibitor combination therapies very frequently induce secondary adrenal insufficiency

**DOI:** 10.1038/s41598-021-91032-6

**Published:** 2021-06-02

**Authors:** Katsunori Manaka, Junichiro Sato, Maki Takeuchi, Kousuke Watanabe, Hidenori Kage, Taketo Kawai, Yusuke Sato, Takuya Miyagawa, Daisuke Yamada, Haruki Kume, Shinichi Sato, Takahide Nagase, Taroh Iiri, Masaomi Nangaku, Noriko Makita

**Affiliations:** 1 Department of Nephrology and Endocrinology, Graduate School of Medicine, The University of Tokyo, 7-3-1 Hongo, Bunkyo-ku, Tokyo, 113-8655 Japan; 2 Department of Respiratory Medicine, Graduate School of Medicine, The University of Tokyo, Tokyo, Japan; 3 Department of Urology, Graduate School of Medicine, The University of Tokyo, Tokyo, Japan; 4 Department of Dermatology, Graduate School of Medicine, The University of Tokyo, Tokyo, Japan; 5 Department of Pharmacology, St. Marianna University School of Medicine, Kawasaki, Japan

**Keywords:** Diseases, Endocrinology, Medical research, Oncology

## Abstract

Immune checkpoint inhibitors (ICIs) are potent therapeutic options for many types of advanced cancer. The expansion of ICIs use however has led to an increase in immune-related adverse events (irAEs). Secondary adrenal insufficiency (AI) can be life-threatening especially in patients with delayed diagnosis. We retrospectively investigated secondary AI in ICI-treated patients. A total of 373 cancer patients treated with ICIs were included and evaluated. An adrenocorticotropic hormone (ACTH) deficiency was described in 13 patients. Among 24 patients with a combination of nivolumab and ipilimumab therapy, 7 patients (29%) developed secondary AI in a median time of 8 weeks during the combination therapy and 2 of 15 patients (13%) developed isolated ACTH deficiency during maintenance nivolumab monotherapy following the combination therapy. More than half of the patients (4/7) with a combination therapy-induced multiple anterior hormone deficiencies was diagnosed as secondary AI based on regular ACTH and cortisol tests with slight subjective symptoms. **S**econdary AI can arise frequently and rapidly in cancer patients receiving a combination ICI therapy, and thus we speculate active surveillance of AI using regular ACTH and cortisol tests during the combination therapy might be useful for avoiding life-threatening conditions due to secondary AI.

## Introduction

ICI-induced endocrine dysfunction can involve the thyroid, parathyroid, pituitary, adrenal gland, and the pancreas^1–5^. Although Immune checkpoint inhibitor (ICI) therapies are often discontinued in patients with severe immune-related adverse events (irAEs) in non-endocrine organs such as the lung, colon, liver and kidney, patients with irAEs involving endocrine organs can continue their ICI regimens if adequate hormone replacement therapy is given^4–10^. Some endocrine disorders such as ACTH deficiency caused by pituitary irAEs (i.e. secondary adrenal insufficiency [AI]) and fulminant type 1 diabetes mellitus (DM), are life-threatening conditions, especially when the diagnoses are delayed. For the early diagnosis of type 1 DM during ICI use, patient education, routine glucose monitoring, and self-measurements of blood glucose levels are recommended^11^. In the case of secondary AI however, in which the ACTH levels cannot appropriately increase against the low levels of cortisol, most presenting symptoms such as fatigue, appetite loss, and joint pain are non-specific, often resulting in a delayed diagnosis^1–3,12^.

In our current study, we addressed some of the pertinent clinical questions regarding the adequate diagnosis of secondary AI induced by ICI therapies in daily clinical practice. We suspected whether routine ACTH and cortisol tests might be helpful for the early diagnosis of secondary AI, whether certain symptoms or laboratory data could indicate secondary AI prior to conducting ACTH and cortisol tests, and what might be done to diagnose secondary AI in daily practice. In our current study, we retrospectively surveyed ICI-induced secondary AI in a cohort from our hospital.

## Results

### Baseline profiles of the study subjects

A total of 373 patients (269 men and 104 women) who received ICI therapies at our hospital were included in this study (Table 1). The median age of this cohort was 68 years (range 23–86 years). The cancers targeted by the ICI regimens in this population included head and neck cancer, esophageal cancer, gastric cancer, lung cancer, kidney cancer, bladder cancer and upper urinary tract (renal pelvis and ureter) urothelial carcinoma, malignant melanoma, and other malignancies (malignant pleural mesothelioma, pancreatic cancer, Hodgkin’s lymphoma, and cancer of unknown primary). The patients who received a combination therapy of ipilimumab and nivolumab had stage 4 kidney cancer (renal cell carcinoma) or stage 4 malignant melanoma.

**Table 1:** Number of treated patients and ICI regimens used.

ICI regimens used	Number of treated patients^a^	Age	Sex	Primary site	Prior ICI therapies	Secondary adrenal insufficiency
Nivo	224	Median 66 [23–86] Mean 64.3	M 166 F 58	Stomach 60 Esophagus 29 Kidney 35 Melanoma 25 Lung 23 Head and neck 51 Others 4	None 211	0/211
					Nivo + Ipi 15	2/15
					Pembro 1	0/1
Pembro	119	Median 70 [26–86] Mean 67.9	M 82 F 37	Bladder and upper urinary tract 48 Lung 44 Melanoma 12 Head and neck 10 Kidney 2 Others 4	None 115	2/115
					Ipi 4	1/4
Ipi	8 ^b^	Median 66.5 [42–86] Mean 63.4	M 5 F 3	Melanoma 8	Nivo 7	1/7
					Pembro 1	0/1
Anti-PD-L1 agents	29	Median 74 [48–81] Mean 70.7	M 21 F 8	Lung 27 Merkel Cell carcinoma 2	None 24	0/24
					Nivo 2	0/2
					Pembro 3	0/3
**Nivo + Ipi**	**24**	Median 67 [40–81] Mean 64.7	M 16 F 8	Kidney 16 Melanoma 8	**None 22**	**6/22**
					**Nivo 2**	**1/2**

ICI, immune checkpoint inhibitor; nivo, nivolumab; pembro, pembrolizumab; ipi, ipilimumab, M, male; F, female.

(a) Some patients were treated with multiple regimens and the “Number of treated patients” is therefore greater than the actual number of patients (n = 373).

(b) All 8 cases treated with ipilimumab alone had undergone a first-line therapy with anti-PD-1 antibodies (nivolumab or pembrolizumab).

Twenty-nine of our current study patients had pre-existing hypothyroidism due to chronic thyroiditis, the use of tyrosine-kinase inhibitors, or a thyroidectomy. One patient had hypopituitarism due to a history of craniopharyngioma. Four patients had an adrenal insufficiency due to glucocorticoid use, bilateral adrenal metastases, and a bilateral adrenalectomy. Seventy-two patients were receiving drugs for DM.

### Endocrine immune-related adverse events other than pituitary ones

Although beyond the main focus of this study, we noted that thyroid irAE developed in 37 patients, adrenalitis (primary AI) developed in one patient, and fulminant type 1 diabetes mellitus developed in 3 patients in our current cohort (Table 2).

**Table 2:** Endocrine irAEs and ICI regimens used.

Category	Number of patients	Ongoing use			
		Anti-PD-1 antibody Nivo (total 209) Pembro (total 116)	Anti-CTLA-4 antibody (total 8)	Anti-PD-1 + CTLA-4 antibodies (total 24)	Anti-PD-L1 antibody (total 30)
*Thyroid*	37				
Hypothyroidism	23	18	2		3
Subclinical hypothyroidism	7	5	1		1
Transient thyrotoxicosis	7	5		2	
*Pituitary*	13				
Isolated ACTH deficiency	5	5 (nivo 2, pembro 3)			
Multiple anterior pituitary hormone deficiency	8		1	7	
*Adrenal*	1				
Primary adrenal insufficiency	1	1			
*DM*					
Fulminant type 1 DM	3	2		1	

ACTH, adrenocorticotropic hormone; ICI, immune checkpoint inhibitor; nivo, nivolumab; pembro, pembrolizumab; DM, diabetes mellitus.

### Serious adverse events other than endocrine ones

ICIs were discontinued due to serious irAEs in 60 of our current study patients (a cumulative total of 61 patients). Interstitial pneumonia was the most common irAE resulting in the cessation of ICI treatments (31 patients). We were concerned that the frequency of serious irAEs might increase in the patients with AI due to an acceleration of autoimmune responses. We noted however that the patients with ICI-induced AI showed no statistically significant increase in such severe complications, compared to patients without ICI-induced AI (Fisher’s exact test; *p* = 0.25; odds ratio 2.16 (95% confidence interval 0.48–7.82)).

### Adverse events in the pituitary gland

**Figure 1:**
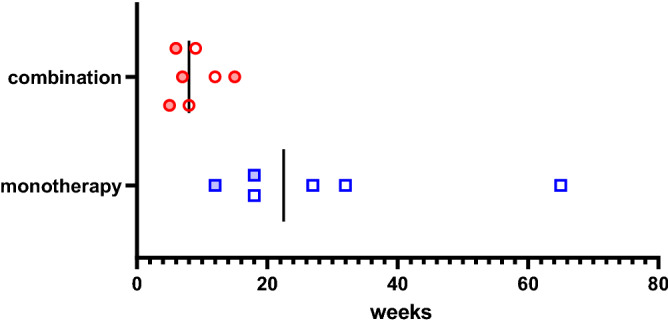
Time to develop secondary adrenal insufficiency (SAI) from initial injection of ICIs in 13 patients. In the combination therapy group, 7 patients developed secondary AI in a median time of 8 weeks during nivolumab and ipilimumab combination therapy. In the monotherapy group, 6 patients developed secondary AI in a median time of 22.5 weeks during nivolumab, pembrolizumab or ipilimumab monotherapy. In the combination therapy group, filled red circles show the timings of SAI diagnosis based on regular ACTH and cortisol tests, and open red circles show the timings of SAI diagnosis based on symptoms. In the monotherapy group, filled blue squares show the timings of SAI diagnosis during maintenance nivolumab monotherapy following the combination therapy, and open blue squares show the timings of SAI diagnosis during pembrolizumab or ipilimumab monotherapy without history of a combination therapy.

**Figure 2:**
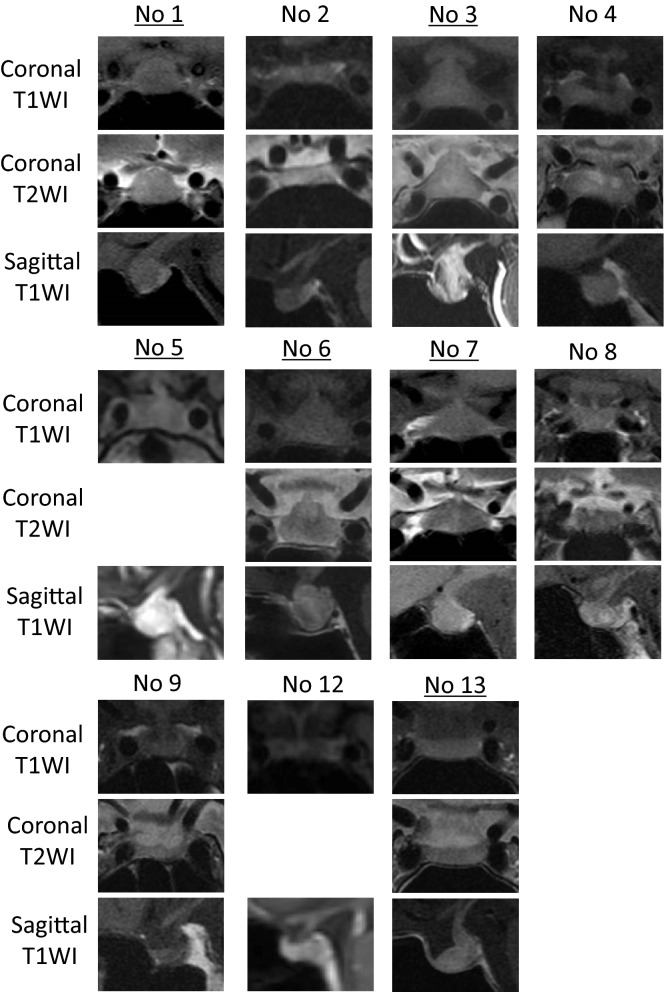
Pituitary MRI of the patients with secondary adrenal insufficiency. MRI analysis revealed enlargement of the pituitary gland in patients No 1, 3, 5–7, and 13 (highlight with underline). Top panels are coronal non-enhanced T1WI, middle panels are coronal T2WI (patents No 5 and 12 lack coronal T2WI), and bottom panels are sagittal enhanced T1 (if not available, non-enhanced T1WI) for each patient.

Secondary AI arose in 13 of the study patients (Tables 1 and 3). Eight of these patients showed multiple anterior pituitary hormone deficiencies, which include ACTH deficiency, and remaining 5 patients showed an isolated ACTH deficiency. Multiple anterior pituitary hormone deficiency occurred in 7 patients who underwent a combination therapy of ipilimumab and nivolumab, and in 1 patient who was administered ipilimumab after nivolumab (Table 1). Isolated ACTH deficiency occurred in 2 patients receiving pembrolizumab alone, 2 patients treated with nivolumab after a combination therapy of ipilimumab and nivolumab, and 1 patient who was administered pembrolizumab after nivolumab alone and ipilimumab alone (Table 1). Hence, 9 of 24 patients (38%) who received a combination therapy of ipilimumab and nivolumab developed secondary AI. It developed during the combination therapy in 7 of these patients (7/24; 29%) and during early courses of maintenance nivolumab therapy after four courses of combination therapy in the 2 remaining patients (Tables 1 and 3, Fig. 1). In contrast, none of our 212 (0%) study patients receiving nivolumab alone without history of the combination therapy, only 3 of our 119 (3%) study patients receiving pembrolizumab alone, and 1 of 8 (13%) patients administered ipilimumab alone developed secondary AI (Table 1). In addition to the high frequency of secondary AI in patients who received the combination therapy, most secondary AI developed during the combination therapy. MRI analysis revealed enlargement of the pituitary gland in 6 of the 8 (75%) patients with multiple anterior hormone deficiency (i.e. hypophysitis) and revealed no enlargement in pituitary gland in any patient with isolated ACTH deficiency (Table 3, Fig. 2). One patient without swelling in the pituitary gland underwent an MRI at 3 weeks after the diagnosis of anterior pituitary hormone deficiency (patient No 4) and the other 7 patients received an MRI within 2 weeks of this diagnosis. Headache symptoms were very characteristic of the 5 patients with pituitary swelling (Fig. 2, patients No. 1, 3, 5–7; 83%).

**Table 3:** Detailed clinical profiles of the patients with ICI-induced secondary AI.

Patient no	Age	Sex	Cancer	At onset of SAI	Weeks at diagnosis	Pattern of secondary AI		Pituitary swelling w/c headache	Symptoms and signs (SS)		Casual levels		Other irAEs
						IAD	Multiple hormone deficiencies		Except headache	Serum sodium (mmol/L)	ACTH (pg/mL)	Cortisol (μg/dL)	
1	50	M	RCC	Nivo + ipi	9		● ACTH, TSH, PRL	● Headache	Fatigue, appetite loss, nausea, eosinophilia	139	3.2	0.3	Transient thyrotoxicosis
2	75	M	RCC	Nivo + ipi	8		● ACTH, Gn, PRL	–	Fatigue, appetite loss, eosinophilia	141	2.7	0.2	
3	40	F	MM	Nivo + ipi	6		● ACTH, TSH	● Headach (continuous)	Eosinophilia	142	15.1	1.0	Fluminant Type 1 DM
4	63	M	MM	Nivo + ipi	5		● ACTH, TSH, Gn	– Headache	Fatigue, eosinophilia	141	1.3	1.4	
5	77	F	MM	Nivo + ipi	15		● ACTH, TSH	● Headache (continuous)	Appetite loss, pain in joints	139	2.0	0.6	
6	81	F	MM	Nivo + ipi	7		● ACTH, TSH, Gn, PRL, GH	● Headache	appetite loss, fatigue	131	4.6	0.5	
7	66	M	MM	Nivo + ipi	12		● ACTH, TSH, Gn, PRL, GH	● Headache	Fatigue, nausea, hypotension	133	2.6	0.3	Enteritis
8	55	M	RCC	Nivo (maintenance Tx)	12	●		–	Hiccup, appetite loss, weakness, hyponatremia	110	1.6	0.6	Diarrhea
9	76	M	RCC	Nivo (maintenance Tx)	18	●		–	Fatigue, appetite loss, eosinophilia	136	3.5	0.3	
10	73	M	Bladder	Pembro	27	●		Not tested	Appetite loss, vomiting,	134	1.2	0.3	
11	74	M	Bladder	Pembro	18	●		Not tested	Septic shock, hyperkalemia, hypoglycemia	134	8.1	2.2	
12	42	M	MM	Pembro	65	●		–	Fatigue, nausea, eosinophilia	137	9.0	2.8	Thyroiditis on ultrasound
13	86	M	MM	Ipi	32		● ACTH, Gn	●	Fatigue, appetite loss, hypoglycemia, eosinophilia, diarrhea	142	1.0	1.3	

Four patients (No 3–6) were diagnosed their SAI based on the results of regular ACTH and cortisol tests.

A TSH deficiency was diagnosed based on TSH, fT4, and/or a thyrotropin-releasing hormone (TRH) loading test.

ACTH, adrenocorticotropic hormone; TSH, thyroid stimulating hormone; Gn, gonadotropin; PRL, prolactin; GH, growth hormone; M, male; F, female; RCC, renal cell carcinoma; MM, malignant melanoma; Bladder, bladder carcinoma; combination, combination therapy of ipilimumab and nivolumab; nivo, nivolumab; pembro, pembrolizumab; ipi, ipilimumab; cs, courses; IAD, Isolated ACTH deficiency; SAI, secondary adrenal insufficiency; SS, symptoms and signs; DM, diabetes mellitus.

**Figure 3:**
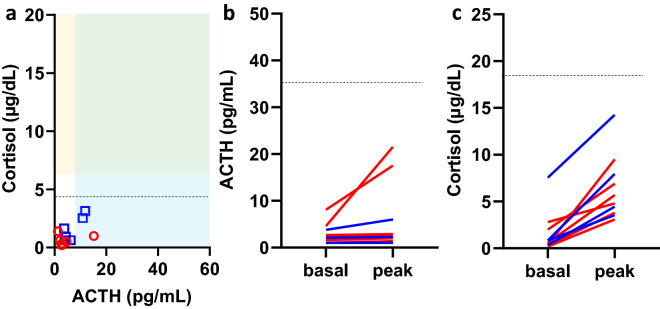
ACTH and cortisol levels upon the suspicion of secondary adrenal insufficiency (AI) and loading test results for the hypothalamus–pituitary–adrenal axis. **(a)** Casual plasma ACTH levels and serum cortisol levels on regular tests or upon the suspicion of a secondary AI are presented in the scatter plot. Open red circles reflect data of patients in the combination therapy group, and open blue squares reflect data of patients in the monotherapy group. The blue zone shows the reference interval for the plasma ACTH level in the morning (7.2–63.3 pg/mL), and the yellow zone shows the interval for the serum cortisol level in the morning (7.07–19.6 μg/dL). Serum cortisol levels were all decreased to below 4.0 μg/dL (dotted line) without elevated plasma ACTH levels. **(b)** A corticotropin releasing hormone (CRH) test was conducted in which synthetic human CRH (100 μg) was intravenously injected and blood samples were collected before injection and at 30, 60, 90, and 120 min afterwards. The baseline and peak ACTH levels are shown. Red lines reflect results of patients in the combination therapy group, and blue lines reflect results of patient in the monotherapy group. The cut-off peak value of ACTH (35 pg/mL) is indicated by the dotted line. **(c)** A rapid ACTH test was performed in which synthetic ACTH (1–24) at 250 μg was intravenously injected. Blood samples were collected prior to the injection and at 30 and 60 min afterwards. The baseline and peak cortisol levels are shown. Red lines reflect results of patients in the combination therapy group, and blue lines reflect results of patient in the monotherapy group. The cut-off peak value of cortisol (18 μg/dL) is indicated by the dotted line.

In our present study cohort, the plasma ACTH levels and serum cortisol levels were measured at every outpatient visit in the patients undergoing combination therapy for malignant melanoma (regular checkup) and when there was a suspicion of AI from symptoms and signs. The casual serum cortisol levels, which were checked regularly or to work up the possibility of secondary AI, were low (2.8 μg/dL at highest) in the morning or under stressful conditions with inappropriately low or normal levels of ACTH (Fig. 3a). To evaluate the pituitary-adrenal axis and diagnose possible secondary AI, loading tests were sequentially performed. Except patient No. 4, 10, and 12, 10 of the 13 patients in our present series diagnosed with an ACTH deficiency using a loading test with corticotropin releasing hormone (CRH), an endogenous ACTH secretagogue, were examined for adrenal function by single ACTH loading. This indicated a reduced cortisol response in all 10 patients (Fig. 3b, c). At first, we suspected that concomitant adrenalitis might have developed in secondary AI patients because a rapid ACTH loading test was performed soon after the ACTH deficiency developed. However, subsequent prolonged ACTH loading tests revealed a preserved cortisol response in 5 of the secondary AI patients (Table 3: patients No. 1–3, 6, and 7) who showed a reduced cortisol response to the single ACTH loading.

### Investigation of factors related to secondary adrenal insufficiency

It would be very clinically useful to be able to diagnose secondary AI based on abnormalities in routine laboratory data. It is well known in this regard that hyponatremia and/or eosinophilia are signs of AI and it has been reported that thyroid stimulating hormone (TSH) deficiency often develops in patients with ipilimumab-induced hypophysitis^13^. As thyrotoxic patients due to thyroid irAEs also show low TSH levels, we investigated TSH levels with free thyroxine (fT_4_) levels. Fisher’s exact tests revealed that new-onset eosinophilia and low TSH levels with normal or low fT_4_ levels were related to secondary AI (odds ratio 6.6 (*p* < 0.01) and 22.1 (*p* < 0.01), respectively) (Table 4). There was no significant association between hyponatremia and secondary AI (Table 4). None of these variables was found to be both highly sensitive and specific for this condition.

**Table 4:** Detailed clinical profiles of the patients with ICI-induced secondary AI.

New-onset	AI		Sensitivity	Specificity	OR (95% CI) ^a^
	+	−			
Eosinophilia					
+	8	63	0.62	0.81	6.6 (1.83–26.5), *P* < 0.01
−	5	261			
Hyponatremia					
+	5	58	0.39	0.81	2.7 (0.68–9.9), *p* = 0.14
−	8	254			
Low TSH levels with normal or low fT4 levels					
+	5	8	0.38	0.97	22.1 (4.64–100.4), *P* < 0.01
−	8	292			

OR: odds ratio, CI: confidence interval.

^a^Fisher’s exact tests were performed to compare the incidence of secondary AI.

## Discussion

We have here retrospectively analyzed cancer patients treated with ICIs at our hospital and found that a combination therapy with ipilimumab and nivolumab can cause secondary AI at a surprisingly high frequency. The incidence of secondary AI was as high as 29% (7/24) in the patients receiving a combination therapy and as 13% (2/15) in the patients under a maintenance therapy just after ICI combination therapy (Table 3). Meanwhile, no patients who received nivolumab therapy without history of the combination therapy (0/212) developed secondary AI. It seems that the frequency of secondary AI during nivolumab maintenance therapy just after the combination therapy was high compared to nivolumab monotherapy. As for time to develop secondary AI, we cannot discuss statistical difference of it between the combination therapy group (median: 8 weeks) and monotherapy group (median: 22.5 weeks) because there may be period bias between routine ACTH and cortisol test patients and non-routine test patients in the combination therapy group (Fig. 1). Based on previous reports showing that the median time to develop pituitary irAEs from initial injection of ICIs is 9 weeks with ipilimumab^14^, 4.9 months with nivolumab and 3.3 months with pembrolizumab^7^, we speculate that a combination therapy might cause pituitary irAEs at least as early as a monotherapy with ipilimumab.

When we focused on the regimens under which ICI-induced secondary AI developed, all of the multiple anterior pituitary hormone deficiencies in our current cohort (8 patients) had developed in patients receiving an anti-CTLA-4 antibody treatment (including ICI combination therapy patients)^15^, and all isolated ACTH deficiencies (5 patients) had developed in patients treated with anti-PD-1 antibody alone (Table 3). This particular pattern of pituitary irAEs (hypophysitis with multiple hormone deficiencies vs. isolated ACTH deficiency) was consistent with the findings of previous studies^16–19^. This pattern is also supported by MRI images (hypophysitis with swelling of pituitary gland vs. isolated ACTH deficiency without swelling; Fig. 2, Table 3).

We speculated on the reasons why secondary AI was frequently diagnosed in our study patients receiving a combination ICI therapy. First, in malignant melanoma patients receiving this combination therapy, routine measurements of ACTH and cortisol levels, in addition to as TSH and fT_4_ levels, had been performed. Notably, 4 patients (patients No 3–6) among the 7 patients who were diagnosed with secondary AI under a combination ICI therapy were diagnosed based on a routine ACTH and cortisol test (Table 3). In the other 3 subjects (patients No 1, 2 and 7), the ACTH and cortisol levels might not have been checked, depending on the extent of the signs and symptoms. Kobayashi et al. also reported that the frequency of ipilimumab-induced hypophysitis was as high as 24% in their prior prospective study^20^. Hence, active surveillance may have contributed to the increased incidence of secondary AI with an ICI combination therapy in our current cohort. Given the likelihood that secondary AI during an ICI combination therapy develops as frequently as thyroid irAEs for which routine monitoring is recommended in guidelines^4,6,9^, active surveillance of AI using regular ACTH and cortisol tests, at least during the combination therapy, may be a desirable and reasonable approach considering the costs and benefits although no clinical guidelines have recommended it so far^4,5,7–10^.

Second, in addition to the increased incidence of secondary AI detected by active surveillance, we suspect that secondary AI may develop more frequently in Japanese patients than previously thought. We speculate that the genetic background of the Japanese population might affect the frequent incidence in pituitary irAE. A pilot study has described human leukocyte antigen (HLA)‐DQB1*06:01, HLA‐DPB1*09:01, and HLA‐DRB5*01:02 as a risk allele for anti-PD-1 antibody induced pituitary irAE, and HLA‐C*03:03 as a protective allele for this condition^21^. Another study has suggested that HLA DR15, B52 and Cw12 may be predictive markers for pituitary irAE^22^. Considering that CTLA-4 expressed in the pituitary gland may be a trigger of ipilimumab-induced hypophysitis via classical complementary activation^15^, a polymorphism in the CTLA-4 gene might be responsible for pituitary irAE, similar to autoimmune thyroid disease^23^.

We paid attention to headache in our patients as they may be an important early indicator of secondary AI caused by hypophysitis with pituitary swelling (Table 3). It has been reported previously that headache and fatigue are the most common presenting symptoms in ipilimumab containing therapy-induced secondary AI^2,19,24,25^. We surmise from our present observations that headache may be more specific to secondary AI whereas fatigue is a non-specific symptom often seen in cancer patients. It has been reported that more than a third of patients with pituitary adenoma complain of headache and that 60% of patients with lymphocytic hypophysitis commonly present with headache before pituitary hormone changes can be characterized^26^. Focusing on the 24 patients who received a combination ICI therapy (Table 1), 6 of the 7 patients with multiple anterior hormone deficiency complained of headache whereas no patient with isolated ACTH deficiency (0/2), nor any of the patients without pituitary hormone dysfunction (0/15), reported this symptom during ICI therapies. In contrast, 5 of 7 patients with multiple anterior hormone deficiency, both patients with isolated ACTH deficiency, and 8 of 15 patients without pituitary hormone dysfunction complained of fatigue during ICI therapies. In addition, continuous headache for several days was noted in 2 patients with hypophysitis before pituitary dysfunction was confirmed using hormonal tests (patients No. 3 and 5). Headache, especially when continuous, may thus be an early specific sign of ICI-induced hypophysitis. Besides headache, we investigated other factors that could be related to secondary AI and that may also be useful early markers of this condition. New-onset eosinophilia and low TSH levels with normal or low fT_4_ levels showed an association with secondary AI, but were neither highly sensitive nor specific factors (Table 4). Hyponatremia, which is also frequently shown in AI, showed no significant association with secondary AI. Hyponatremia had low positive productive value (0.079) and could be caused by many other common conditions, such as infection, appetite loss, and dehydration.

It is known that the function of the cortisol secreting cells in the adrenal gland is maintained by ACTH secreted from the pituitary. It has been reported that when ACTH is deficient for one week after pituitary surgery, cortisol secreting cells start to be functionally suppressed^27–29^. In such patients, the adrenal gland cannot react to single ACTH loading, but is recovered by continuous ACTH loading^12^. In our current cohort, we originally suspected that primary AI onset coincided with that of secondary AI because rapid ACTH loading test performed soon after developing symptoms of AI had indicated a reduced cortisol response (Fig. 3c). However, a subsequent prolonged ACTH loading test indicating a normal cortisol response ruled out the coincidence of primary AI. Based on our present series, low serum cortisol levels (cortisol < 4.0 μg/dL) in the early morning or under a stressful condition with inappropriately low or normal levels of ACTH might have enough diagnostic value for ICI-induced secondary AI. Given that a rapid onset ACTH deficiency in ICI-induced secondary AI may be a risk factor for adrenal crisis, reflected by the occurrence of grade 4 irAEs in two of our present patients (patient No. 11, septic shock; patient No 8, severe hyponatremia; Tables 2 and 3), we again contend that an earlier diagnosis of AI is a key to avoiding life-threatening conditions due to it.

This study had several limitations of note. Principal among these were its retrospective and single center design, small number of included patients, and the heterogeneity of the target cancers and ICIs used. In our cohort, although the number of patients who receive combination therapy of ipilimumab and nivolumab was small, the incidence of secondary AI was much higher than in previous reports. Considering the combination therapy might be approved for additional indications against various cancers, management for a secondary AI will become more important. Our current findings may provide useful information in the meantime that can assist with strategies to reduce severe endocrine-related irAEs.

## Materials and methods

### Subjects

All 373 patients who were treated with immune checkpoint inhibitors (ICIs) at our hospital between 1 April 2014 and 31 July 2020 were retrospectively evaluated. The ICI regimens used in this population included nivolumab (n = 224), pembrolizumab (n = 119), ipilimumab (n = 8), atezolizumab (n = 18), durvalumab (n = 10), avelumab (n = 1), and a combination of nivolumab and ipilimumab (n = 24) (Table 1).

This study was approved by The University of Tokyo Hospital Research Ethics Committee (Comprehensive approval of retrospective studies: authentication number 2879-(7)). Informed consent was obtained in the form of opt-out on the web-site and no written informed consent was required to access the medical records, since this study was a retrospective study without any intervention. This study adheres to the ethical principles outlined in the Declaration of Helsinki as amended in 2013 and the Ethical Guidelines for Medical and Health Research Involving Human Subjects in 2017.

### Data collection

We retrospectively collected the following data for our study patients: age, sex, history of drug use (including ICIs, glucocorticoids, levothyroxine, and glucose lowering agents), cancer type, imaging results (pituitary MRI), concomitant disease, and blood test results (serum sodium, potassium, chloride, calcium, albumin, phosphorus, creatinine, casual plasma glucose, HbA1c, immune reactive insulin (IRI), c-peptide (CPR), antiglutamic acid decarboxylase (anti-GAD) antibody, ACTH, cortisol, TSH, fT_4_, free triiodothyronine (fT_3_), growth hormone (GH), insulin-like growth factor-1 (IGF-1), luteinizing hormone (LH), follicle stimulating hormone (FSH), estradiol, free testosterone, total testosterone, prolactin, plasma renin activity, serum aldosterone concentration, and dehydroepiandrosterone-sulfate (DHEA-S)). All results of blood tests were collected from baseline (before ICI injection) to last administration course of ICI.

### Definitions of hyponatremia, eosinophilia, low TSH, and low fT_4_

Hyponatremia was defined as a serum sodium concentration lower than 135 mmol/L and eosinophilia as a count of not less than 500 eosinophils per microliter of blood. TSH was measured with enzyme immunoassay using ST AIA-PACK TSH (TOSOH, Ltd., Tokyo, Japan) on AIA-2000 (TOSOH, Ltd., Tokyo, Japan). FT_4_ was measured with fluorimetric enzyme-linked immunoassay using ST AIA-PACK FT4 (TOSOH, Ltd., Tokyo, Japan) on AIA-2000 (TOSOH, Ltd., Tokyo, Japan). Low TSH was defined as TSH value below the lower limit of the reference range (TSH reference range 0.38–4.31 μIU/mL) and low fT_4_ was defined as fT_4_ value below the lower limit of the reference range (fT_4_ reference range 0.82–1.63 ng/dL).

### Methods and assessments of loading tests

#### Corticotropin-releasing hormone (CRH) loading test

One ampule of corticorelin (human, 100 μg, Nipro ES Pharma Co., Ltd., Osaka, Japan) was intravenously injected and ACTH and cortisol levels were measured at baseline, and at 30, 60, 90, and 120 min. The plasma ACTH level at peak after loading corticorelin was increased to 2–3-fold higher than baseline and also above 35 pg/mL in patients with normal pituitary function (normal response). A cortisol level above 18 μg/dL at peak after loading coticorelin revealed that the patient had a normal adrenal function in addition to a normal pituitary function.

#### Rapid ACTH loading test

One ampule of tetracosactide acetate (250 μg, Daiichi Sankyo Company, Ltd., Tokyo, Japan.) was administered intravenously and the cortisol levels were measured at baseline, and at 30 and 60 min. The serum cortisol level after loading tetracosactide acetate was generally higher than 18 μg/dL (normal response). A reduced cortisol response (< 18 μg/dL) to tetracosactide acetate indicated the possibility of either primary or secondary AI.

#### Prolonged ACTH loading test

One ample of tetracosactide acetate zinc suspension (500 μg, Daiichi Sankyo Company, Ltd., Tokyo, Japan.) was administered intramuscularly once per day over 3 consecutive days and 24-h urine free cortisol levels and serum cortisol levels were measured at baseline and after administration. The cut-off value for a diagnosis of a reduced adrenal response was a lower than 2-fold increase in 24-h urine free cortisol excretion, compared to baseline, after three administrations of tetracosactide.

### Differential diagnosis of adrenal insufficiency using a prolonged ACTH loading test

A prolonged ACTH loading test enables a differential diagnosis of AI through the response of cortisol. A no/low response of cortisol in this test indicates primary adrenal deficiency induced by adrenal damage. A positive response of cortisol however indicates secondary AI induced by an ACTH deficiency.

### Statistical analysis

The relationships between AI (13 patients of secondary AI and one patient of primary AI, i.e. adrenalitis) and other serious irAEs were examined using a chi-squared test. All statistical analysis was performed using R 3.5.2 software (The R Foundation of Statistical Computing; www.r-project.org). A *p* value < 0.05 was considered to indicate statistical significance.

## Data Availability

The datasets generated during and/or analyzed during the current study are available from the corresponding author on reasonable request.
